# Changes in Soil Bacterial and Fungal Community Composition and Functional Groups During the Artificial Restoration of Degraded Grassland of “Black‐Soil Mountain”

**DOI:** 10.1002/ece3.70361

**Published:** 2024-10-03

**Authors:** Lele Xie, Yushou Ma, Yanlong Wang, Yuan Ma, Xiaoli Wang

**Affiliations:** ^1^ Qinghai Academy of Animal and Veterinary Science Qinghai University Xining Qinghai China; ^2^ Academy of Animal and Veterinary Science Qinghai University Xining Qinghai China; ^3^ Key Laboratory of Alpine Grassland Ecosystem in the Three‐River‐Source, Ministry of Education Xining Qinghai China

**Keywords:** artificial planting, black‐soil mountain, carbon and nitrogen, function group, soil microbial community

## Abstract

About 35% of grassland in Sanjiangyuan area of China has degenerated into black‐soil mountain. Artificial grassland is considered to be an effective measure to alleviate the severely degraded grassland in the alpine region of the three rivers and has been widely used. However, the pattern, potential function, and changes of carbon and nitrogen contents of soil microorganisms in degraded grassland in Heimushan by planting artificial grassland are still unclear. In this study, mixed‐sown artificial alpine grassland (AG) was the focus of our study, whereas degraded black‐soil mountain grassland (BG) and natural alpine grassland (NG) served as controls. Illumina 16S and ITS gene sequence analyses were used to analyze the community structure of the soil bacteria and fungi. The functional groups of NG, AG, and BG were predicted using the FAPROTAX and FUNGuild databases. In addition, the levels of soil carbon, nitrogen, and soil enzyme activities were evaluated. The results indicated a significant increase in the aboveground biomass of BG due to the planting artificial grassland. Moreover, the contents of total carbon (TC), total nitrogen (TN), ammonium nitrogen (${\mathrm{NH}}_{4}^{+}-N$), microbial biomass carbon (MBC), microbial biomass nitrogen (MBN), and leucine aminopeptidase (LAP) increased in the soil. Planting artificial grasslands changed the composition of bacterial and fungal communities. Among these, the bacterial community was more sensitive to planting artificial grasslands. The relative abundance of bacterial functional groups involved in carbon and nitrogen cycling changed significantly, suggesting that bacteria may play a role in regulating nutrient cycling during artificial grassland planting. Soil TC, TN, LAP, and ${\mathrm{NH}}_{4}^{+}-N$ affected the microbial community structure related to carbon and nitrogen. ${\mathrm{NH}}_{4}^{+}-N$ and β‐1,4‐glucosidase were carbon and nitrogen factors, respectively, that affected functional changes in fungi. These results indicate that planting artificial grasslands can effectively enhance the productivity of degraded black‐soil mountain and regulate soil microbial communities and soil physical and chemical properties.

## Graphical abstract

The formation of degraded grassland in black‐soil mountain has attracted the attention of many scholars, and the establishment of artificial grassland is the most effective way to control the extremely degraded alpine meadow—“black‐soil mountain.” In order to better understand the response of degraded grassland to artificial restoration measures, we evaluated the interaction between vegetation and soil microorganisms during artificial restoration of degraded grassland in the black‐soil mountain.

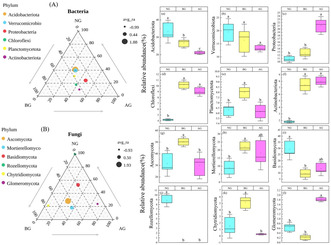

## 1 Introduction

The three‐river source region is located in the southeastern region of the Qinghai–Tibet Plateau. The dominant ecosystem in this region is alpine meadows, which have a great carbon sequestration potential (Zhao et al. 2023a, 2023b). However, in recent years, with the intensification of land use and changes in the natural environment, some alpine meadows have degraded, such as the surface has shown dark brown bald spots, forming secondary bare land black‐soil beaches, and some black‐soil beaches have spread to hillsides or hilltops, forming black‐soil mountain (Dai et al. 2021). The appearance of degraded grasslands in black‐soil mountain leads to a reduction in dominant species, plant productivity, and ecosystem function (Peng et al. 2018; Peng, Cai, and Yu 2018). The emergence of secondary bare land on black‐soil beaches is the most challenging issue in terms of ecological restoration. Restoration or reconstruction of degraded grassland ecosystems and their functions has attracted great interest from many researchers (Nam et al. 2021; Shang and Long 2007; Sun et al. 2024). Conventional management measures include grazing exclusion and fencing to restore degraded black‐soil mountain grasslands (Yin et al. 2021) and reseeding with native grass species (Dong et al. 2010). Although these measures have effectively restored vegetation cover, productivity, and soil nutrients in degraded grasslands, their effects are variable (Tang, Chen, and Yang 2021). In this context, artificial grasslands with *Elymus nutans*, *Poa crymophila Qingha*i, and *Festuca sinensis Qinghai* have been established in Dari County, Guoluo Prefecture, Qinghai Province as an alternative approach.

Previous preliminary studies on the Qinghai–Tibet Plateau have shown that artificial grassland planting is an important method for restoring degraded grasslands in black‐soil mountain and that perennial planting artificial grassland is beneficial for improving carbon and nitrogen storage and carbon and nitrogen concentrations in the alpine meadow Qinghai–Tibet Plateau (She et al. 2023; Wen et al. 2018). Planting artificial grasslands can quickly restore degraded areas, improve plant communities and soil properties, enhance soil carbon sinks, promote vegetation succession, boost forage yield, and reduce grass–livestock conflicts (Li et al. 2023). However, when perennial grasses are used to create artificial grasslands, reverse succession occurs in the grassland community, including soil reverse succession, typically 5–8 years after planting, as a result of excessive grazing pressure (Wang et al. 2022). To solve this problem, extensive research has been conducted on grassland vegetation and soil restoration (Gao et al. 2019; Li et al. 2024; Shang et al. 2008). Restoring ecosystem functions and soil characteristics takes a long time, depending on the time required to restore the associations between different community components (Zhang et al. 2018). Vegetation may not be the best indicator of true recovery success, and it is essential to evaluate the entire system, including microbial relationships (Jiang et al. 2024). Therefore, restoring grassland landscapes to their natural and original state is crucial. A more comprehensive approach to restoration involves examining its relationship with microbial communities. It is essential to determine the properties of plants and soil that affect microbial communities in grasslands.

Soil microorganisms play a crucial role in plant–soil nutrient transformation, organic carbon and nitrogen cycling, pollutant degradation, and other soil biochemical reactions (Sun et al. 2024). A richer and more diverse soil microbial biomass leads to higher soil enzyme activity, highlighting the significance of soil microorganisms in organic matter transformation (Mencel, Mocek‐Płóciniak, and Kryszak 2022). The quantity, activity, and population structure of soil microorganisms may change significantly during vegetation restoration (Chen et al. 2018). Some studies have shown that the diversity of bacterial and fungal communities in highly degraded meadows differs significantly from that in non‐degraded meadows (Li et al. 2016). After the restoration of degraded grasslands, a significant increase in bacterial community diversity has been reported, which is mainly influenced by vegetation type in the restored areas (Hou et al. 2019). The various growth modes of different vegetation types have different effects on soil ecosystems, leading to distinct changes in the soil during different stages of vegetation restoration (Toju et al. 2013). Soil bacteria and fungi play crucial roles in soil biochemical processes and form complex interspecific systems (Wu et al. 2021). Interactions between microbial groups can be negative (competition) or positive (cooperation), affecting the microbial community structure and ecosystem function (Freilich et al. 2010). Understanding the composition and diversity of microorganisms and their interactions is essential for exploring soil function during grassland restoration in degraded black‐soil mountains.

To date, no research has systematically quantified the response of soil bacterial and fungal characteristics to grassland degradation and artificial restoration in black‐soil mountain. Little is known about the relationship between soil microbial communities and soil quality in different grassland types. This study was conducted in a degraded alpine grassland and an artificially restored grassland for the comprehensive management of black‐soil mountain in the three‐river source region of the Qinghai–Tibet Plateau in China. This study offers a unique opportunity to study bacterial and fungal communities. This study aimed to explore the composition and artificial planting. Microbial 16S and ITS gene sequencing were used to assess the effects of artificial planting methods on sof these communities afteril characteristics and microbial indicators in degraded black‐soil mountain grasslands. Based on these objectives, we made the following assumptions regarding alpine grassland. we expect that (1) the aboveground vegetation and soil characteristics will change significantly after the artificial restoration of degraded grasslands in black‐soil mountain; (2) we anticipate to observe differences in soil carbon and nitrogen levels, microbial communities, and functional groups between degraded grassland, natural grassland, and artificially planted grassland in black‐soil mountains; (3) we aimed to identify the primary carbon and nitrogen factors affecting the bacterial and fungal communities in the degraded grassland of black‐soil mountain after artificial restoration.

## 2 Materials and Methods

### 2.1 Study Area

**FIGURE 1:**
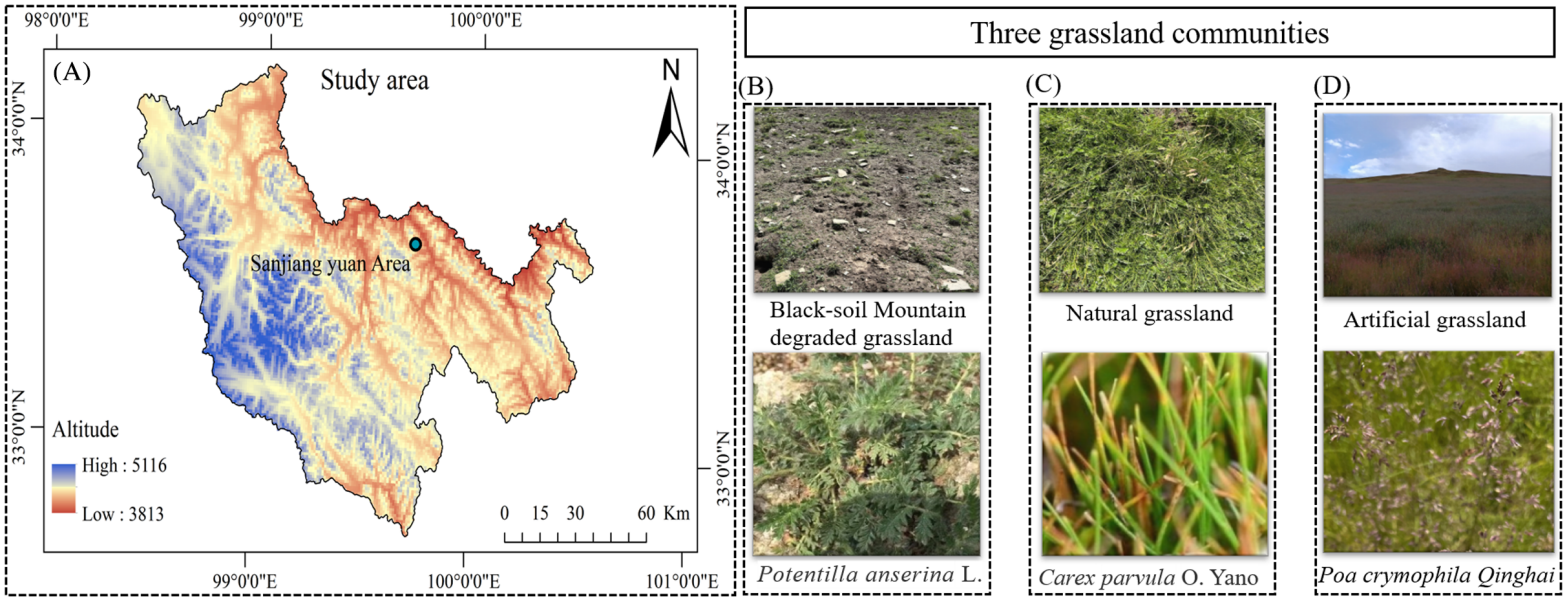
The study area and sampling point (A). Community of three grassland: Black‐soil mountains degraded grassland dominant with *Potentilla anserina* L. (B), natural grassland with *Carex parvula* O. Yano (C), artificial grassland with *Poa crymophila* Qinghai (D).

The study was carried out in an experimental area of artificially restored degraded grassland located in the black‐soil mountain region of the three‐river source region (Figure 1A). Zhiquegou, Wosai Township, Dari County, Qinghai Province (33°40′40″ N, 99°42′59″ E; 4000 m above sea level). The region has a continental plateau climate with harsh climatic conditions, including a cold climate and no absolute frost‐free periods. The average annual temperature ranges from −3.5°C to −0.1°C, with a cold season lasting 8 months. In the coldest month, January, the average temperature is −12.9°C, whereas in the warmest month, July, it is 9.1°C. The region experiences rain and heat during the same season, with an annual average of 2466.5 h of sunshine. The soil types of natural grassland are mainly alpine grass felt soil and black felt soil, the vegetation types are mainly alpine meadows, and the dominant species is Cyperaceae. The pH of the soil was 6.07 (Xie et al. 2023).

The classification of grassland degradation levels involves assessing the indices, types, and classifications of degraded grasslands on black‐soil beaches (Dong et al. 2015). The severely degraded natural grasslands form black‐soil mountain, dominated by broad‐leaved edible grasses (Figure 1B), such as *Pomatosace filicula* Maxim, *Potentilla anserina* L, *Bistorta vivipara* L, and *Cirsium souliei* (*Franch. Mattf*, *Meconopsis integrifolia* (Maxim.) *Franch*).

The natural grassland sample plot was an alpine meadow near a hillside, and the grazing level and management measures were consistent with those of the artificial grassland. Natural grassland is mainly dominated by *Kobresia pygmaea*, *K. humilis*, and *K. capillifolia*. *Poa crymophila*, *Carex atrofusca*, and *Polygonum viviparum* were the companion species (Figure 1C).

We conducted a 5‐year study on grassland restoration in an experimental area of artificial grassland in Dari County. We selected *Elymus nutans*, *Poa crymophila* Qinghai, and *Festuca sinensis* Qinghai as grass species with a mixed sowing ratio of 1.8:2.2:0.5. The sowing amount was 67.5 kg hm^−2^. The amount of fertilizer applied was 300 kg hm^−2^ (total N (18%) + P_2_O_5_ (12%) + K_2_O (5%) ≥ 35.0%) (Figure 1D).

### 2.2 Experimental Design

This experiment was conducted in August 2023 during the peak pasture growth season. Plant community surveys and soil sample collection were also conducted. Representative artificial grassland (AG), natural grassland (NG), and degraded black‐soil mountain grassland (BG) were selected as experimental plots, with three replicates per plot. In each treatment plot, 1 m × 1 m quadrats were randomly set with a distance of no < 5 m between them. The species and names, cover, and height of all the plants within the quadrats were documented. Aboveground biomass within the quadrats was harvested at ground level, weighed for fresh weight, stored in clean envelopes, and transported back to the laboratory. The samples were dried at 65°C and subsequently weighed to determine their dry weights, resulting in a total of nine plant samples. The plant community richness was assessed by determining the total number of plant species present in each quadrat. A soil auger (inner diameter, 3.5 cm) was used to collect five topsoil samples (0–10 cm) from each quadrat following an S‐shaped route, which were combined to form a composite soil sample. This procedure was repeated three times, yielding nine soil samples. Freshly collected soil samples were sieved through a 2‐mm sieve to remove plant roots and debris. The soil samples were immediately transported to the laboratory under cold conditions and divided into two parts: One part was used for soil chemical property analysis, and the other part was stored at −80°C. The latter was sent to Genidnovo Biotechnology Company in Guangzhou for soil bacterial and fungal sequence analyses.

### 2.3 Carbon and Nitrogen Properties of Soil

The concentrations of total carbon (TC) and total nitrogen (TN) in soil were determined using carbon and nitrogen analyzer (FLASHSMART, Germany). Soil nitrate‐nitrogen (${\mathrm{NO}}_{3}^{-}-N$) and ammonia nitrogen (${\mathrm{NH}}_{4}^{+}-N$) levels were determined using potassium chloride leaching (Cai et al. 2017). Soil microbial biomass carbon (MBC) and microbial biomass nitrogen (MBN) were assessed using the fumigation‐extraction method (Yin et al. 2021). The activities of the soil enzymes, β‐1,4‐glucosidase and leucine aminopeptidase (LAP), which are associated with carbon and nitrogen acquisition, were determined using the microplate fluorescence method (Liu et al. 2023).

### 2.4 Bioinformatics Analyses

The composition and diversity of bacteria and fungi in soil microbial communities were examined using a high‐throughput gene assay. Total genomic DNA was extracted from 0.25 g soil samples using a HiPure Stool DNA kit for each sample (Zhao et al. 2023a, 2023b). DNA quality was evaluated using a NanoDrop 2000, and the integrity of the nucleic acid samples was verified through agarose gel electrophoresis. The V4 region of bacterial 16S rRNA was amplified using a specific primer with a barcode. The primer sequences used were 515F: GTGYCAGCMGCCGCGGTAA and 806R: GGACTACNVGGGTWTCTAAT (Parada, Needham, and Fuhrman 2016). Primers for the internal transcribed spacer (ITS) in fungal rRNA, ITS1‐F: CTTGGTCATTTAGAGGAAGTAA and ITS2:GCTGCGTTCTTCATCGATGC, were used for PCR amplification. The amplified products were fragmented, purified, and quantified using a QuantiFluorTM (TM) fluorometer. The purified amplified products were mixed in equal proportions, ligated to sequencing adapters, and used to construct a sequencing library. Finally, the sequences were processed using the Illumina PE250 sequencing technology. To ensure data reliability and validity, FASTP was used to filter the reads from the original dataset generated by the Illumina MiSeq platform. Additionally, FLASH was used to merge paired‐end reads into contigs and remove low‐quality sequences, resulting in the generation of high‐quality contigs (Magoč and Salzberg 2011). The sequencing data were then compared with the UNITE (16S and ITS) databases to retrieve the taxonomic information. The α‐ and β‐diversity indices of bacteria and fungi were calculated at the OTU level, including the Sobs, Chao 1, Simpson, and Shannon indices.

### 2.5 Statistical Analyses

SPSS 21.0 software was used to perform a one‐way ANOVA, with the least significant difference method (LSD) used for multiple comparisons. The significance level was set at *p* < 0.05. After variance analysis, multiple comparisons (LSD method) were used to assess the significance of variances in vegetation, microbial diversity index, and soil enzyme activities. Principal coordinate analysis (PCoA) based on the Bray–Curtis distance was used to evaluate the percentage variation in the bacterial community structure. ADONIS was used to test the significance of differences among various grasslands, whereas ANOSIM analysis was used to detect differences within and between groups. Linear discriminant analysis (LDA) was used to assess the microbial communities and classify different vegetation types based on microbial composition. The LEfSe analysis involved conducting a Kruskal–Wallis rank sum test on all groups, followed by the Wilcoxon rank sum test to compare selected species and genera between the two groups. Finally, LDA was used to select discrepancies, and the results were sorted by mapping to trace evolutionary branches. The Mantel test was used to explore the correlation between soil physical and chemical properties and bacterial and fungal diversity.

## 3 Results

### 3.1 Effects of Artificial Planting on Aboveground Vegetation and Soil Properties of Degraded Grasslands in Black‐Soil Mountains

Variations in vegetation characteristics and soil carbon and nitrogen content were significantly different among the different grasslands (Table 1). The coverage and biomass of the NG and AG areas were significantly higher than those of the BG (*p* < 0.05). Plant richness in NG was significantly higher than that in BG and AG (*p* < 0.05). The soil TC, MBC, TN, ${\mathrm{NH}}_{4}^{+}-N$, and LAP contents in BG were significantly lower than those in NG and AG (*p* < 0.05). Conversely, the levels of BG and ${\mathrm{NO}}_{3}^{-}-N$ in BG were significantly higher than those in NG and AG (*p* < 0.05). The MBN content in AG was significantly higher than that in BG and NG, with no significant difference in the MBN content between BG and NG. In general, BG, related to the carbon cycle, decreased after artificial recovery, whereas LAP, related to the nitrogen cycle, increased significantly after treatment.

**TABLE 1:** Vegetation characteristics and changes of carbon and nitrogen.

		BG	NG	AG
	Coverage (%)	37.33 ± 1.45c	94.00 ± 0.58a	72.33 ± 1.45b
	Biomass (g m^−2^)	102 ± 4.51c	256.67 ± 12.02b	462.13 ± 14.57a
	Species richness	12.00 ± 2.08b	25.33 ± 1.45a	6.67 ± 0.88b
Carbon	TC (g kg^−1^)	39.30 ± 0.44c	48.47 ± 2.69b	60.24 ± 0.98a
	MBC (mg kg^−1^)	1454.86 ± 54.89b	1774.52 ± 88.61ab	2040.22 ± 134.90a
	BG (μmol g^−1^d^−1^)	63.33 ± 3.33a	47.05 ± 3.44b	51.08 ± 1.63b
Nitrogen	TN (g kg^−1^)	3.60 ± 0.03a	4.40 ± 0.20a	5.45 ± 0.08a
	MBN (mg kg^−1^)	63.10 ± 0.80b	62.33 ± 13.32b	116.28 ± 0.93a
	${\mathrm{NH}}_{4}^{+}-N$ (mg kg^−1^)	6.25 ± 1.23b	12.99 ± 1.13a	15.72 ± 1.69a
	${\mathrm{NO}}_{3}^{-}-N$ (mg kg^−1^)	13.99 ± 1.46a	4.54 ± 1.84b	7.14 ± 0.91b
	LAP (μmol g^−1^ d^−1^)	3.84 ± 0.33c	8.26 ± 0.43b	11.33 ± 0.32a

*Note:* AG, artificial grassland the same below; BG, degraded black‐soil mountain grassland; NG, natural alpine grassland. TC, MBC, BG, TN, MBN, ${\mathrm{NH}}_{4}^{+}-N$, ${\mathrm{NO}}_{3}^{-}-N$, and LAP represent the abbreviations of soil total carbon, soil microbial biomass carbon, β‐1,4‐glucosidase (C acquisition enzyme), soil total nitrogen, soil microbial biomass nitrogen, soil ammonium nitrogen, soil nitrate nitrogen, and leucine aminopeptidase (N acquisition enzyme), respectively. Values are the means ± SE (*n* = 3). Lowercase letters indicate significant differences among different grassland types (*p* < 0.05).

### 3.2 OTU Value, Diversity, and Community Composition of Soil Microorganisms in Degraded Grasslands of Black‐Soil Mountains by Artificial Planting

**FIGURE 2:**
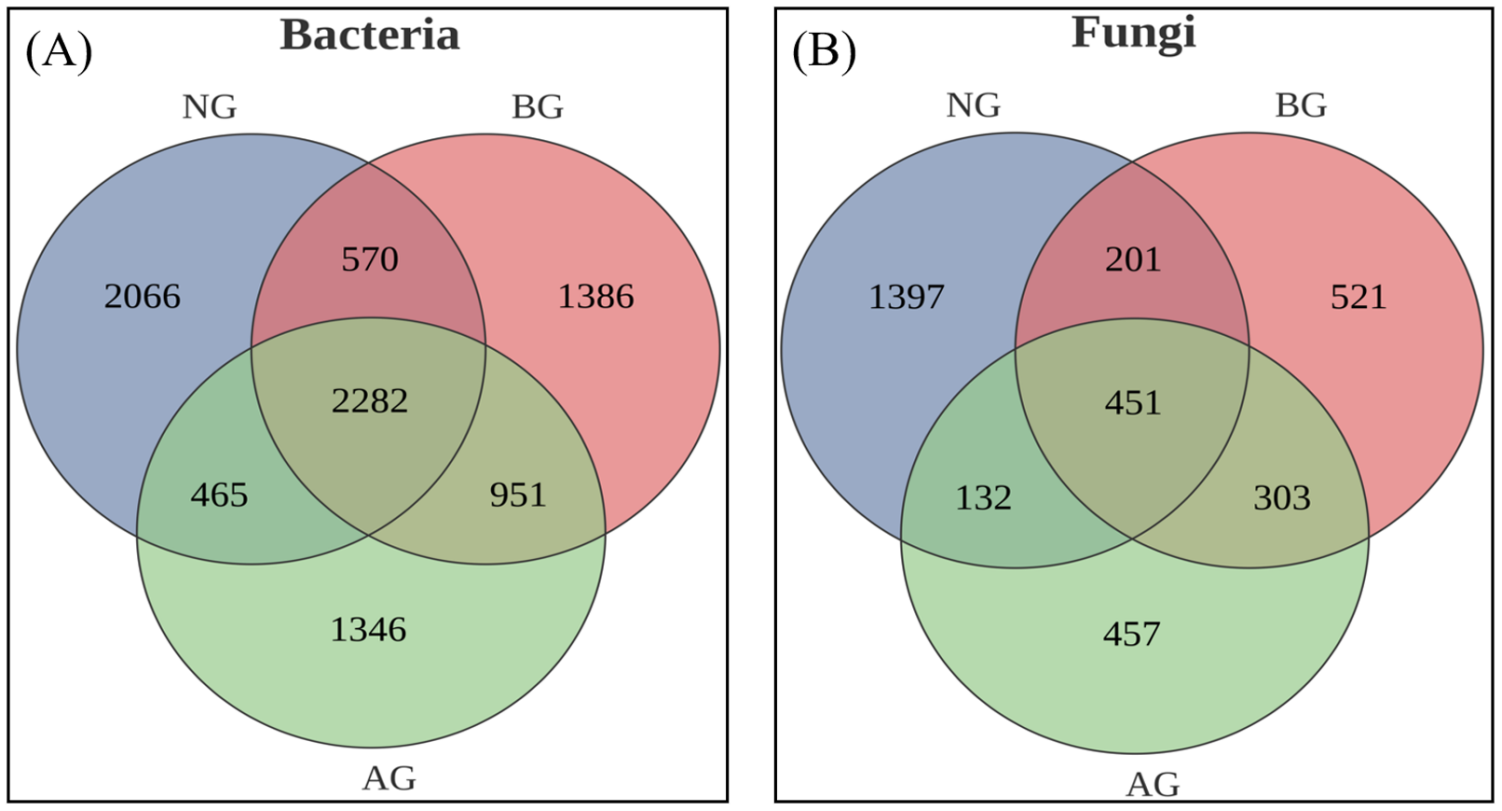
The OTU values of bacteria and fungi in three grasslands. (A) Venn analysis of bacteria OTU, (B) Venn analysis of fungi OTU.

The number of bacterial OTU obtained from NG, BG, and AG was 5383, 5189, and 5044, respectively. A significant increase in bacteria‐specific OTUs was observed in NG compared with BG (Figure 2A), whereas the OTU in AG showed a decreasing trend. However, the numbers of fungal OTU in BG, NG, and AG were 1476, 1343, and 2181, respectively. The number of unique fungal OTUs in BG, NG, and AG was 521, 1397, and 457, respectively. Compared with BG and NG, the number of fungal OTU in AG significantly increased by 705 and 838, respectively (Figure 2B).

**FIGURE 3:**
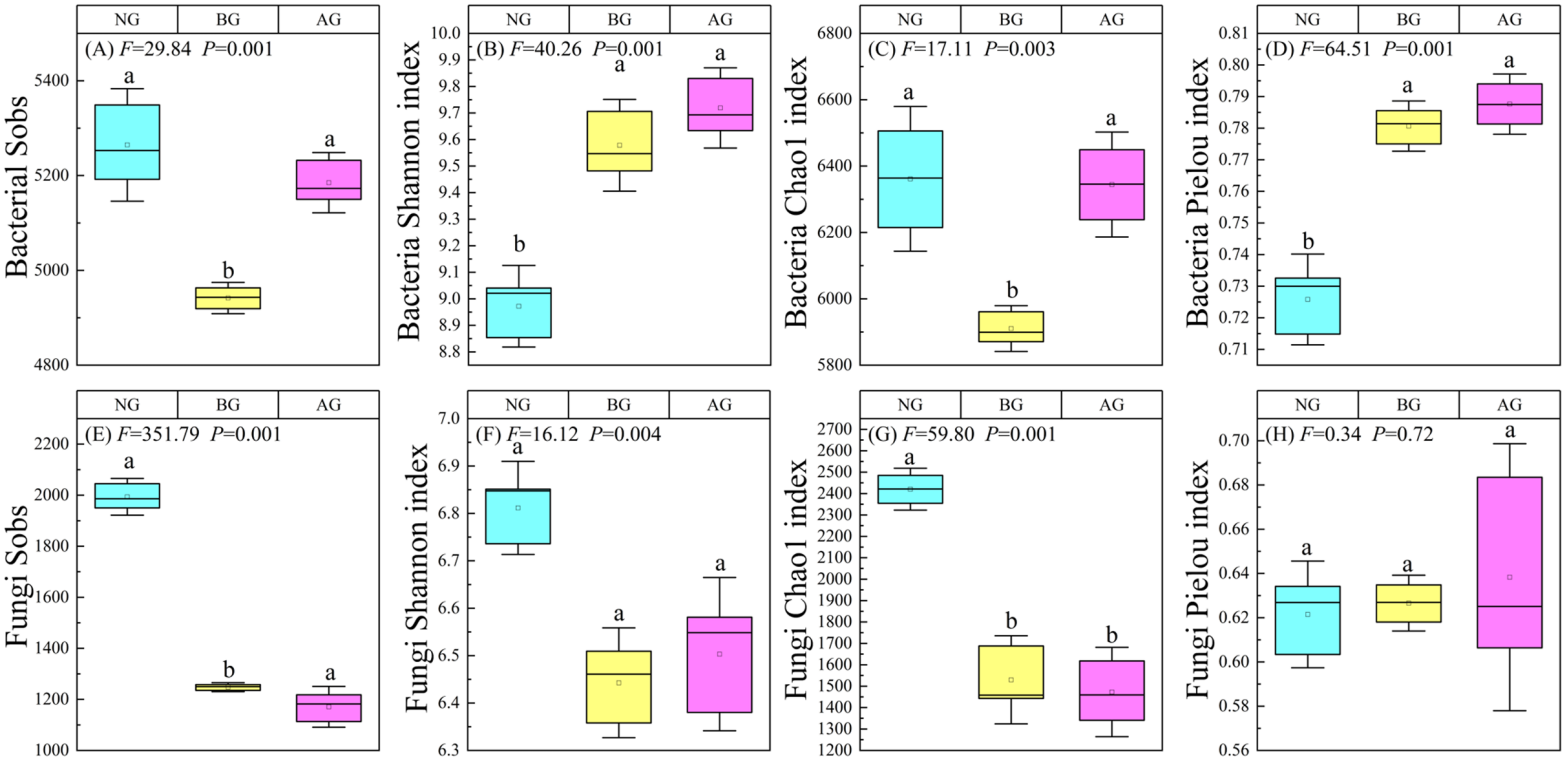
Changes of α diversity of bacteria and fungi in three grasslands. Sobs (A), Shannon index (B), Chao 1 index (C), and Pielou index (D) of bacteria. Sobs (E), Shannon index (F), Chao 1 index (G), and Pielou index (H) of fungi.

There were significant differences in bacterial diversity among the three grasslands (Figure 3, *p* < 0.05). The Sobs index and Chao1 index of NG and AG were significantly higher than those of BG (Figure 3A,C), whereas the Shannon and Pielou indices of NG were significantly lower than those of BG and AG (Figure 3B,D). The Sobs and Chao1 index of fungal diversity in NG were significantly higher than those in BG and AG (Figure 3E,G) (*p* < 0.05). However, there were no significant differences between the Shannon and Pielou index among the three grassland types.

**FIGURE 4:**
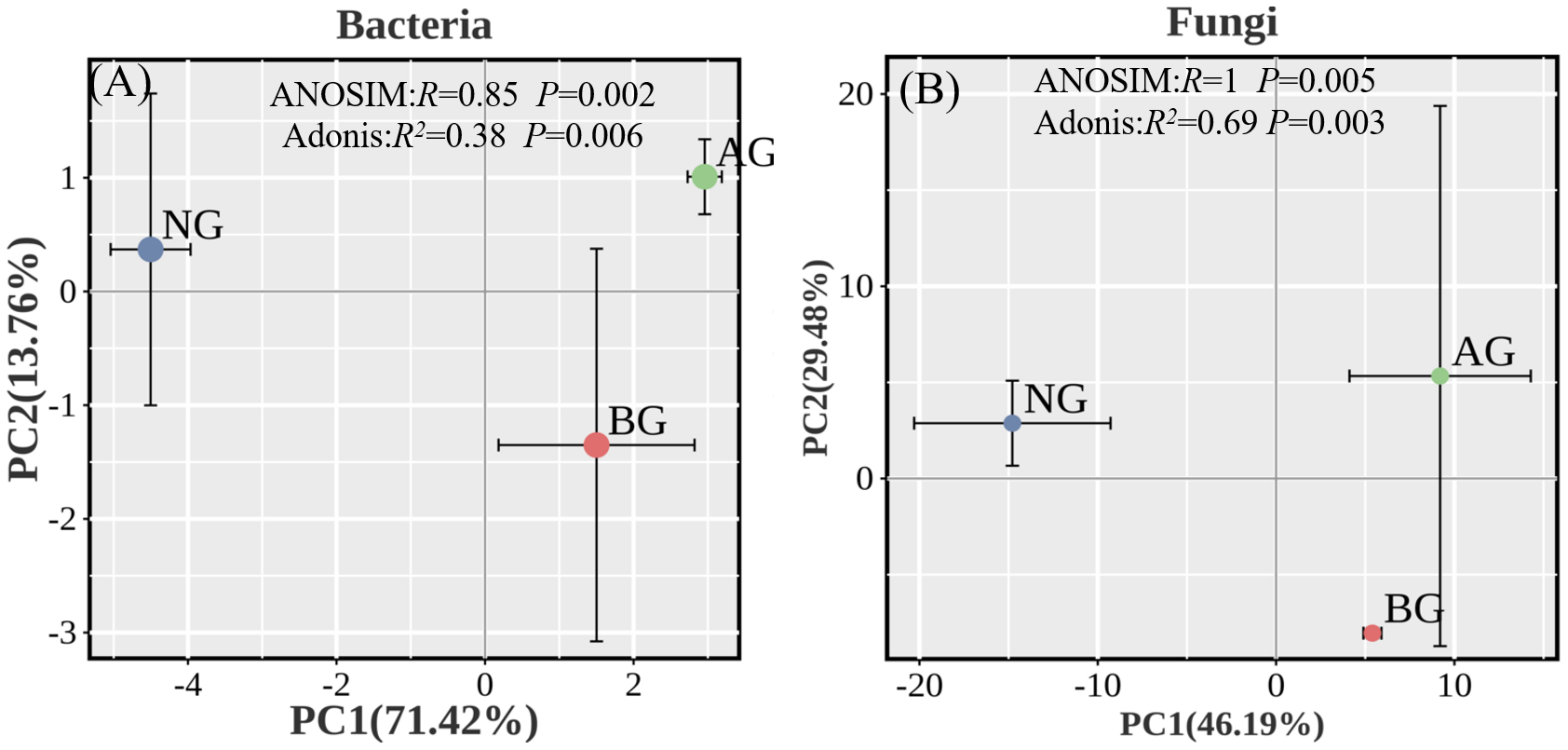
Changes in β‐diversity of bacteria (A) and fungi (B) in three grasslands.

Nonmetric multidimensional scaling (PCoA) based on the Bray–Curtis distance a revealed distinct separation of the community across different grasslands (Figure 4). Subsequent ANOSIM testing (Bray–Curtis) showed a significant dissimilarity in community composition between different grasslands, indicating significant variations in the bacterial and fungal community structures. In addition, the results of the ADONIS test showed that the bacterial and fungal communities changed significantly among grasslands (Figure 4A,B).

### 3.3 Effects of Artificial Planting on Soil Microbial Community Composition of Degraded Grasslands in Black‐Soil Mountains

**FIGURE 5:**
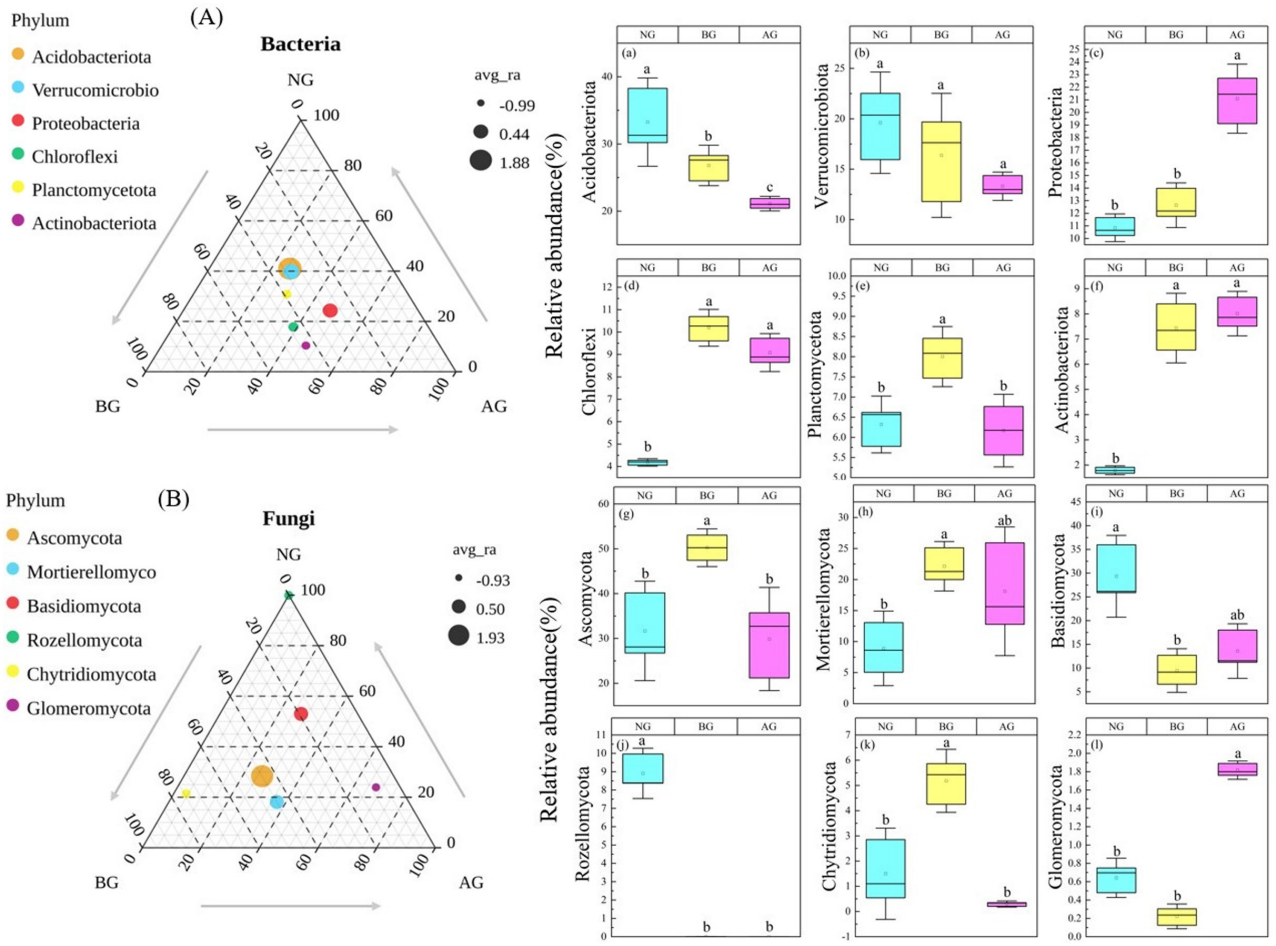
The relative abundance (%) of the soil bacteria (A) and fungi (B) communities in three grassland at the phylum level. The Acidobacteriota (a), Verrucomicrobiota (b), Proteobacteria (c), Chloroflexi (d), Planctomycetota (e), Actinobacteriota (f), Ascomycota (g), Mortierellomycota (h), Basidiomycota (i), Rozellomycota (j), Chytridiomycota (k), Glomeromycota (l).

The vast majority of bacteria in the different grasslands were Acidobacteriota, Verrucomicrobiota, and Proteobacteria. Among them, the relative abundance of Acidobacteriota was the highest, exceeding 20% in different locations. Acidobacteriota and Verrucomicrobiota tended to decrease compared with BG and NG (Figure 5A). The dominant fungi in different grasslands were Ascomycota, Mortierella, and Basidiomycota. In addition, Rozellomycota, Chytridiomycota, and Glomeromycota were present in most soils but their relative abundance was low (Figure 5B). In BG, the relative abundance of Ascomycota was significantly higher than that in NG and AG (Figure 5g), whereas that of Mortierellomycota was significantly higher than that in NG, but there was no significant difference compared with AG (Figure 5b). In NG, the relative abundance of Rozellomycota tended to increase (Figure 5i), which was significantly higher than that in BG, but there was no significant difference with AG, whereas the relative abundance of Rozellomycota was significantly higher than that in BG and AG (Figure 5j).

**FIGURE 6:**
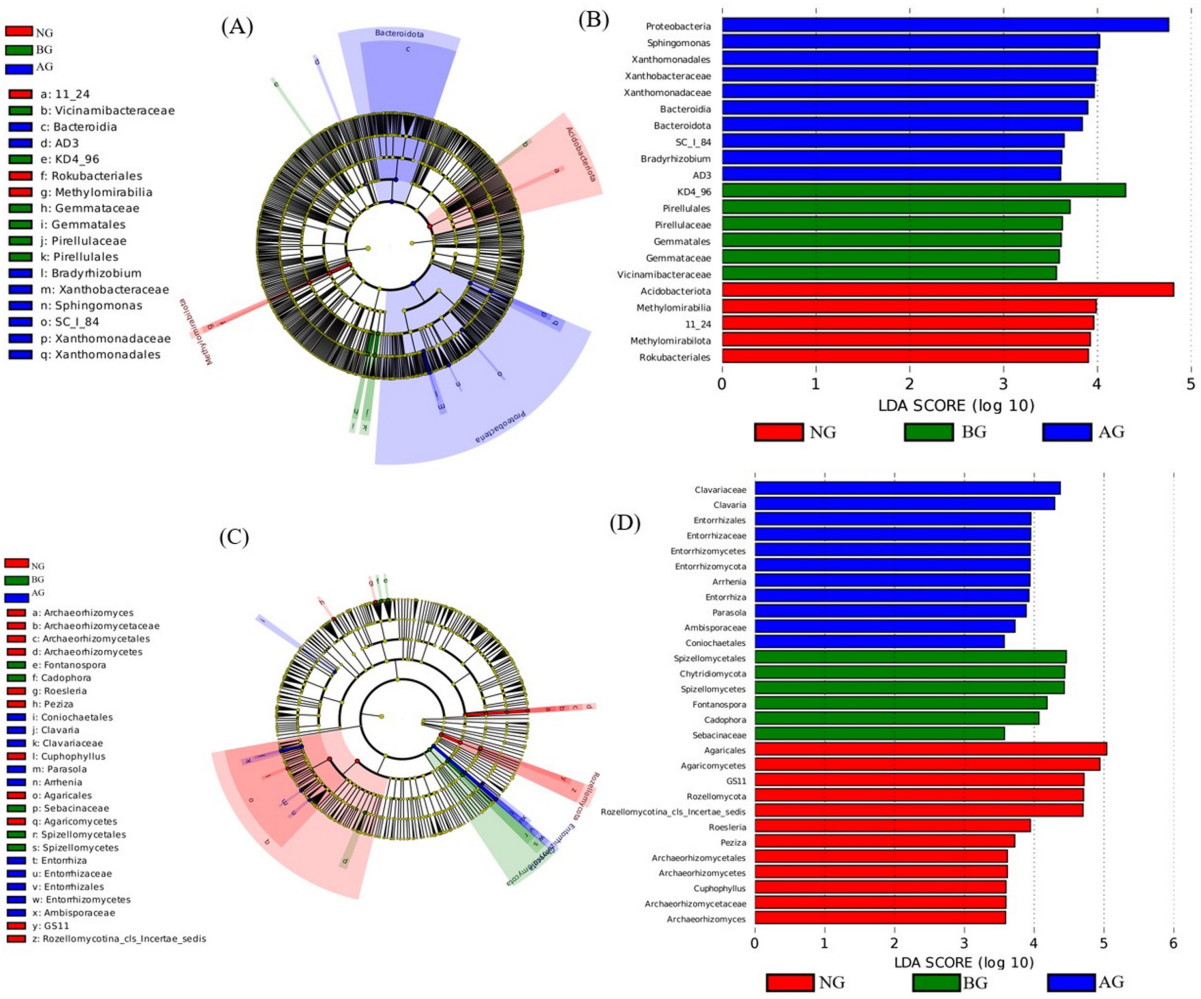
The cladogram shows significant differences between bacteria (A) and fungi (C) enrichment groups. LDA score chart shows biomarker in different grassland and the length of the histogram represents the influence of different species (B, D). The groups with significant differences in abundance between different grassland are represented by colored dots, and cladogram circles represent phylogenetic taxa from phylum to genus. Only the LDA scores > 4 of bacteria and fungi were shown.

Lefse analysis showed that the fungal community changed significantly after AG plantation in the BG. Specifically, at the phylum, class, and genus levels, certain bacterial groups were significantly enriched at different locations (Figure 6A). Most bacterial groups were predominantly found in the AG; for example, Proteobacteria, Sphingomonas, Xanthomonadales, and Xanthobacteraceae increased significantly in the AG (Figure 6A,B). Fungal groups were mainly concentrated in NG; for example, Agaricales and Rozellomycota increased significantly in NG (Figure 6C,D).

### 3.4 Effects of Artificial Planting on Functional Groups of Soil Microbial Communities in Degraded Grasslands of Black‐Soil Mountains

**FIGURE 7:**
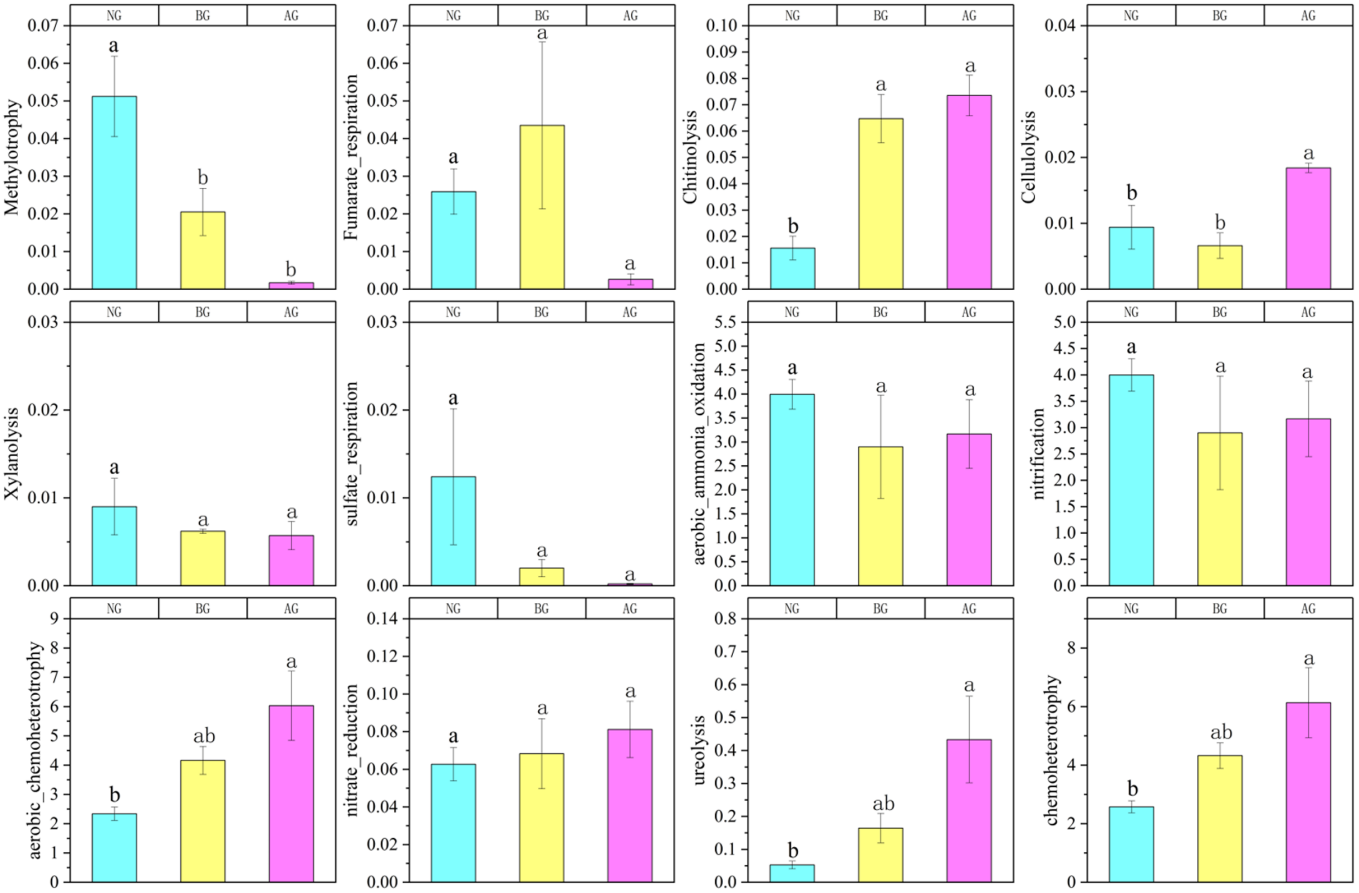
Changes of soil bacterial functional groups in different grassland. Different letters indicate significant differences between treatments (*p* < 0.05).

Functional labeling of soil bacterial communities was performed based on the FAPROTAX database. NG, BG, and AG were divided into 66, 56, and 44 functional groups, respectively, which were mainly involved in the biochemical processes of carbon and nitrogen cycling. Functional groups related to nitrogen cycling included aerobic chemoheterotrophy and chemoheterotrophy, nitrification, aerobic_ammonia_oxidation, nitrate_reduction, and ureolysis. The functional groups related to the carbon cycle included methylotrophy, fumarate_respiration, chitinolysis, cellulolysis, xylanolysis, and sulfate respiration. Significant changes in other functional groups related to C and N cycling were observed in AG compared with NG. For instance, chitinolysis and cellulolysis, which are associated with carbon cycling, significantly increased. Additionally, there were increasing trends in nitrification, aerobic chemoheterotrophy, nitrate reduction, ureolysis, and chemoheterotrophy related to nitrogen cycling (Figure 7).

**FIGURE 8:**
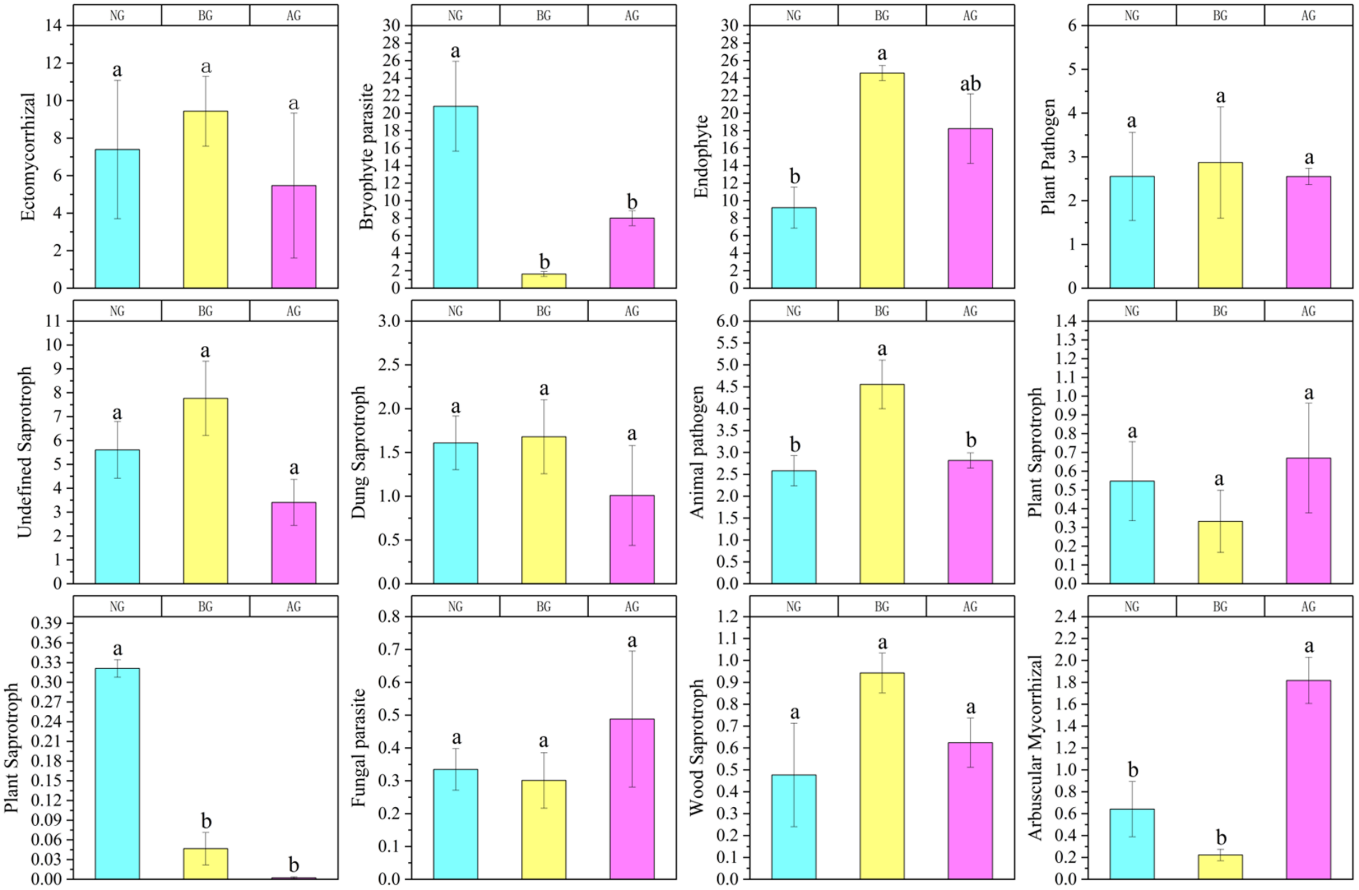
Changes of soil fungi functional groups in different grassland. Different letters indicate significant differences between treatments (*p* < 0.05).

We used the FUNGuild database to annotate fungi and provide three types of nutritional models: symbiotrophs, saprotrophs, and pathotrophs. The results showed that the number of symbiotic and pathogenic organisms in BG increased rapidly, and FUNGuild obtained more detailed information regarding the nutritional model from the selected soil (Figure 8). The relative abundance of ectomycorrhizal fungi in BG was high, but the difference was not significant compared with that in NG and AG. Compared with BG, bryophyte parasites, plant saprotrophs, fungal parasites, and arbuscular mycorrhizal fungi were significantly increased in AG. However, regarding ectomycorrhizal, undefined saprotroph, dung saprotroph, and wood saprotroph, no significant differences were observed in the NG, BG, and AG.

### 3.5 Carbon and Nitrogen Factors Affect Microbial Community Diversity, Structure, and Function

**FIGURE 9:**
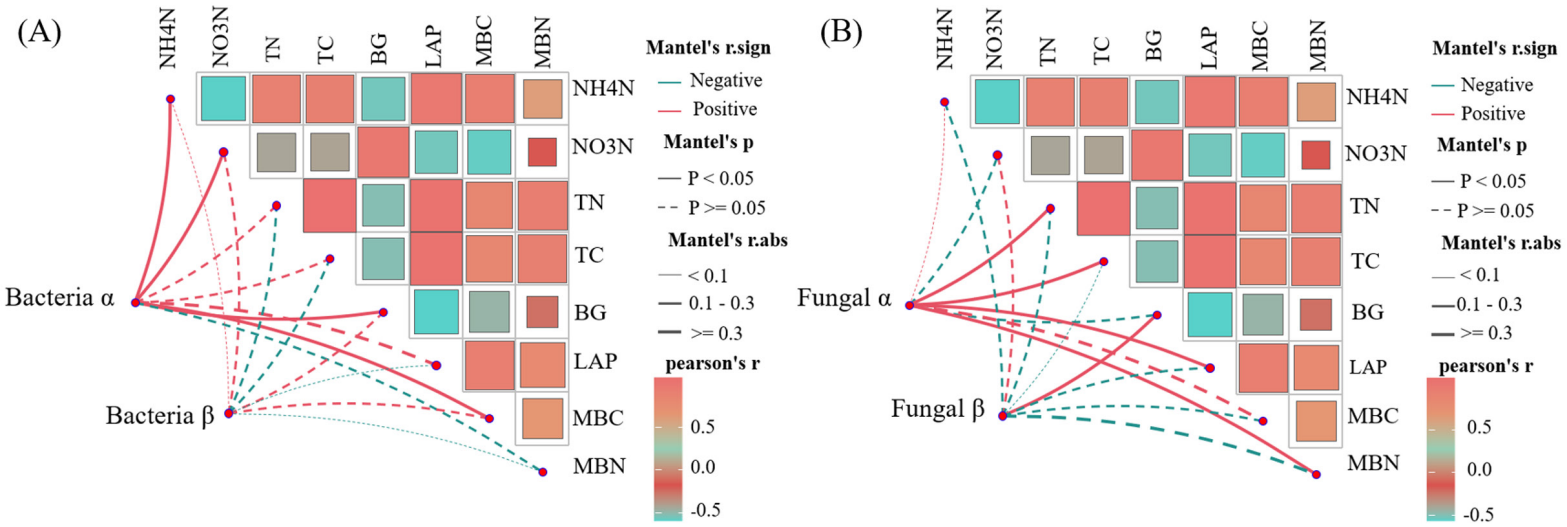
Evaluation of diversity index of bacteria (A) and fungi (B) related to soil physical and chemical properties by Mantel test.

The Mantel test revealed a positive correlation between the observed α‐diversity of bacteria and ${\mathrm{NH}}_{4}^{+}-N$, ${\mathrm{NO}}_{3}^{-}-N$, BG, and MBC (Figure 9A). Additionally, the α‐diversity of fungi was positively correlated with TN, TC, LAP, and MBN (Figure 9B). Moreover, fungal β‐diversity was positively correlated with BG activity.

**FIGURE 10:**
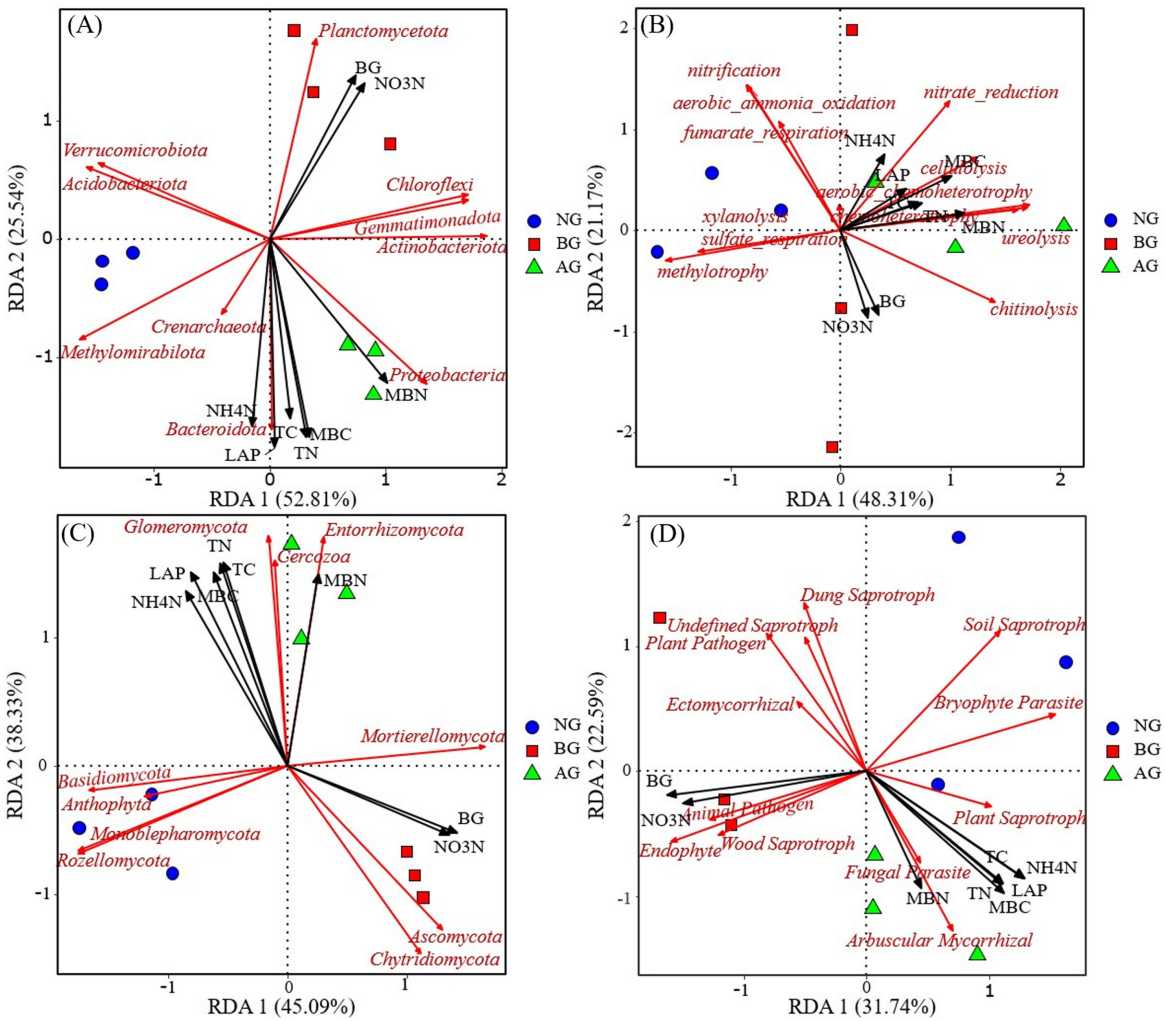
Ordination plots of the results from the redundancy analysis (RDA) to identify the relationships between the bacterial and fungal community and carbon, and nitrogen (A, C) and to identify the relationships between the bacterial and fungal functional group with the carbon and nitrogen characteristics (black arrows) (B, D). TC, MBC, BG, TN, MBN, ${\mathrm{NH}}_{4}^{+}-N$, ${\mathrm{NO}}_{3}^{-}-N$, and LAP represent the abbreviations of soil total carbon, soil microbial biomass carbon, β‐1,4‐glucosidase, soil total nitrogen, soil microbial biomass nitrogen, soil ammonium nitrogen, soil nitrate nitrogen, and leucine aminopeptidase, respectively.

On the first two axes, RDA explained 78.35% and 83.42% of the relationship between bacterial (Figure 10A), fungal (Figure 10C), and soil factors, respectively. Similarly, RDA explained the relationship between the functional groups of bacteria (Figure 10B), fungi (Figure 10D), and soil factors by 69.48% and 54.33%, respectively. In addition, our results showed that LAP, TN, and TC were the most important soil factors affecting the bacterial and fungal community structure (*p* < 0.001, *p* < 0.01, Table 2). Soil factors had no significant influence on the structure of bacterial functional groups, but the main factors influencing the structure of fungal functional groups were BG, LAP, and ${\mathrm{NH}}_{4}^{+}-N$ (*p* < 0.01, *p* < 0.05, Table 3). Changes in MBC were also closely related to changes in bacterial and fungal community structure and fungal function (*p* < 0.01, *p* < 0.05).

**TABLE 2:** Characteristics of influencing factors of horizontal change of soil microbial phylum.

Environmental factor	Bacteria		Fungi	
	*R* ^2^	*p*	*R* ^2^	*p*
${\mathrm{NH}}_{4}^{+}-N$	0.79	0.01	0.79	0.02
${\mathrm{NO}}_{3}^{-}-N$	0.74	0.03	0.66	0.04
TN	0.91	0.003	0.86	0.004
TC	0.90	0.004	0.86	0.003
BG	0.76	0.017	0.71	0.03
LAP	0.96	0.001	0.90	0.004
MBC	0.72	0.016	0.81	0.004
MBN	0.78	0.029	0.71	0.04

**TABLE 3:** Characteristics of influencing factors on changes of soil microbial functional groups.

Environmental factor	Bacteria		Fungi	
	*R* ^2^	*p*	*R* ^2^	*p*
${\mathrm{NH}}_{4}^{+}-N$	0.22	0.48	0.74	0.01
${\mathrm{NO}}_{3}^{-}-N$	0.25	0.41	0.71	0.02
TN	0.19	0.52	0.63	0.05
TC	0.18	0.55	0.63	0.04
BG	0.26	0.39	0.83	0.009
LAP	0.16	0.59	0.73	0.01
MBC	0.41	0.22	0.68	0.02
MBN	0.40	0.21	0.33	0.28

## 4 Discussion

### 4.1 Changes in Carbon and Nitrogen During Artificial Restoration of Degraded Grasslands in Black‐Soil Mountains

Many studies have been conducted on the influence of planting forage on soil characteristics, which have proven that artificial planting has advantages in improving soil TC and TN (Li et al. 2021). We found that the soil TC, TN, ${\mathrm{NH}}_{4}^{+}-N$, and ${\mathrm{NO}}_{3}^{-}-N$ contents showed an increasing trend compared with BG (Table 1). This is because fertilization in AG increases the input of plant residues produced by plant roots into the soil, and the soil structure tends to be stable, thus increasing the contents of TC and TN. Second, the primary productivity of AG was higher than that of BG, which is more beneficial for plants to provide rich organic matter to soil (Li et al. 2014). Therefore, artificial grassland planting can effectively promote the formation and accumulation of soil carbon and nitrogen.

Climate and the physical and chemical properties of soil are key factors that influence extracellular enzyme activity (Cao et al. 2016). The results of our study revealed that the LAP content in AG was higher than that in BG and NG (Table 1). This may be attributed to the higher productivity of the plant community in AG compared with BG, which is conducive to the better survival of soil fauna and microorganisms. In addition, the soil TC, TN, ${\mathrm{NH}}_{4}^{+}-N$, and ${\mathrm{NO}}_{3}^{-}-N$ were higher in the artificial grassland than in the degraded grassland, indicating a rapid turnover of soil carbon and nitrogen and increased accumulation. Consequently, this provides substrates for LAP, which plays a role in the carbon and nitrogen cycles. This finding was consistent with the conclusions of Xiao et al. (2023). In contrast, BG content in degraded grasslands was higher than that in AG and NG. This can be attributed to a more complex vegetation community structure and higher soil fauna and microbial activity in the degraded grassland, thereby enhancing the activity of the carbon and nitrogen cycle enzymes in the degraded alpine meadow. This shows that the biochemical reaction rates of carbon and nitrogen in alpine meadows were faster than those in the AG and NG. This is because alpine meadows have more favorable environmental conditions, a relatively complex vegetation community structure, and higher activities of soil fauna and microorganisms, all of which promote the activities of carbon and nitrogen cycling enzymes in degraded alpine meadows (Zhao et al. 2023a, 2023b; Wang et al. 2022).

The distribution characteristics of soil MBC and MBN in alpine soils are influenced by various factors, such as climate, vegetation, soil physicochemical properties, and nutrient content (Sapkota et al. 2021). In this study, the content of MBC and MBN increased gradually in AG and decreased gradually in BG. This finding is consistent with that of Hao (2010). Our results showed that MBC and MBN accumulated during the process of artificial restoration, indicating that planting artificial grassland can increase microbial biomass, soil fertility, and microbial activity. Furthermore, the study area is situated on the Qinghai–Tibet Plateau, where climate variability leads to significant differences in water availability, resulting in uneven vegetation growth. Increased precipitation can stimulate mineralization and the accumulation of soil nutrients in the ecosystem (Niu et al. 2008). Soil microorganisms can accumulate high levels of microbial biomass carbon and nitrogen in response to increased nutrient availability in the soil. This indicated that AG significantly enhanced soil activity, leading to improved microbial respiration, activity, and biomass. Consequently, this enhances carbon and nitrogen utilization by microorganisms, resulting in a significant increase in MBC and MBN. Therefore, planting artificial grassland contributed to vegetation restoration, microbial biomass accumulation, and soil quality.

### 4.2 Changes in Soil Microbial Diversity and Community Composition During Artificial Restoration of Degraded Grasslands in Black‐Soil Mountains

In this study, the success of artificial planting in the restoration of BG was investigated from the perspective of the microorganisms. Generally, restoration success is gauged by the attainment of the target plant species community and near‐complete vegetation coverage. However, an increasing number of studies have shown that plants serve as only one indicator of restoration success, with soil microbial communities offering more information for the restoration of ecosystem functions (Zhao et al. 2019). Changes in microbial community composition are often closely related to changes in ecosystem function mediated by them (Allison et al. 2013). Therefore, understanding the mechanisms of soil microbial communities is imperative to understand how ecosystem processes respond to environmental changes (Freedman et al. 2015). The dominant phylum of the bacterial community was consistent across the different plots; however, their relative abundances varied significantly, with distinct responses observed among the dominant groups (Figure 5A). In this study, the bacterial communities in different soil samples were mainly composed of Acidobacteriota, Verrucomicrobiota, Proteobacteria, Chloroflexi, Planctomycetota, and Actinobacteriota. This finding is consistent with that of Zhao et al. (2019) for the AG of the Qinghai–Tibet Plateau. Actinomycetes can decompose refractory organic matter, such as cellulose and chitinolysis, thereby improving the soil carbon cycle (Eilers et al. 2010). Proteobacteria can produce extracellular polysaccharides, which contribute to the formation of biological crust and soil stability and are associated with phenolic and aromatic compounds in the soil (Nam et al. 2021). Acidobacteria and chloromycetes play complementary roles in ecosystems. Acidobacteria are primarily involved in the breakdown of organic matter to supply energy and nutrients to other microorganisms, whereas Chlorobiota are primarily responsible for carbon fixation through photosynthesis (Klatt et al. 2013). Compared with BG, Acidobacteria, Verrucomicrobia, and Planctomycetes exhibited an increasing trend in the AG (Figure 5a,b,e); however, Proteobacteria, Chloroflexi, and Actinobacteriota exhibited a decreasing trend in AG (Figure 5c,d,f). The varied response patterns of the dominant groups at different locations were primarily attributed to distinct environmental factors that govern the formation of microbial communities in each region. Surprisingly, Acidobacteriota and Verrucomicrobiota were the most abundant bacteria in alpine meadows. This phenomenon confirmed the adaptability of Acidobacteriota and Verrucomicrobiota to the soil habitat conditions of AG, which was also supported by a recent study on grassland ecosystems (Chai et al. 2019). Fungal communities were mainly composed of Ascomycota, Mortierellomycota, Basidiomycota, Rozellomycota, Chytridiomycota, and Glomeromycota (Figure 5B) and this finding is consistent with the findings of Li et al. (2016). Ascomycetes and Basidiomycetes are the main fungi that play an important role in apoplastic decomposition and nutrient cycling and participate in the carbon cycle (Unterseher, Peršoh, and Schnittler 2013). Fungal Ascomycetes usually inhabit harsh environments, whereas Basidiomycetes prefer resource‐rich environments with a wide variety of plants. This discrepancy explains the significant variation in the relative abundance of ascomycetes between the study plots and the relatively high presence of Basidiomycetes in NG and AG (Nara 2008). In addition, compared with BG, Mortierellomycota and Glomeromycota exhibited an increasing trend in AG (Figure 5h,l); however, Basidiomycota and Chytridiomycota showed a decreasing trend (Figure 5i,k), possibly because of the cold, poorly permeable soil environment formed on the Tibetan Plateau under unique natural conditions (Peng et al. 2018; Peng, Cai, and Yu 2018). In addition, with changes in the original geological landform and soil physical and chemical properties, soil microorganisms have formed distinct groups during natural evolution to adapt to the interference of human factors (such as reckless land reclamation, extensive management, and indiscriminate mining and excavation of wild resources). Therefore, it is speculated that environmental stress influences the microbial community composition. Overall, in the extreme climate environment of the Qinghai–Tibet Plateau, grassland soil microorganisms, bacteria, and fungi have developed a unique community composition that collectively resists external environmental pressure and maintains microbial community stability. However, further research is required to fully understand these interactions.

A change in the aboveground vegetation type causes a change in soil factors, resulting in variations in bacterial and fungal diversity (Yarwood and Högberg 2017). In this study, the OTU numbers of bacteria and fungi in NG were higher than those in AG and BG (Figure 2), which was consistent with the findings for soil microorganisms in AG (Yang et al. 2015). The Sobs and Chao1 index of bacterial diversity and Sobs, Shannon, and Chao1 index of fungi exhibited increasing trends in the NG. This finding was consistent with those of Wu et al. (2021), who demonstrated that grasslands with high plant diversity often exhibit high microbial diversity. One explanation is ecological niche complementarity; plant diversity can increase the variety and abundance of microorganisms in the soil by providing more diverse resources to soil microorganisms (Steinauer, Chatzinotas, and Eisenhauer 2016). The formation of soil fungal communities is associated with the characteristics of various plant species (Cline and Zak 2015). In this study, the dominant species in NG, AG, and BG exhibited distinct characteristics and established different niches (Herz et al. 2017). However, plant communities produce organic matter that restricts growth through litter changes, thereby limiting the microbial biomass and affecting fungal diversity. Compared with BG, the vegetation composition in AG increased by planting *Elymus nutans*, *Poa pratensis* cv. Qinghai, and *Poa pratensis* cv. Qinghai. This modification resulted in variations in the quality and quantity of fungal decomposition and utilization, which affected the soil fungal community. Thus, the greater functional diversity of plant traits in AG shapes the soil fungal community, potentially contributing to the decrease in the BG fungal diversity.

According to the PCoA results, the bacterial and fungal community structures varied among the different grasslands (Figure 4). This finding is consistent with previous studies that reported significant differences in microbial communities in the alpine grasslands of the Qinghai–Tibetan Plateau during degradation or restoration succession (Guo et al. 2019). The interaction between microorganisms was enhanced during the restoration of the AG. This enhancement in microbial interactions during AG restoration could be attributed to the increased presence of soil components in the restored grasslands, leading to an improvement in soil quality (Gao et al. 2021).

Analysis by Lefse provides novel insights into the responses of soil bacteria and fungi to dynamic changes in the soil environment (Zhao et al. 2023a, 2023b). In this study, most bacterial groups (Proteobacteria, Sphingomonas, Xanthomonadales, and Xanthobacteraceae) were predominantly found in the AG (Figure 6B), indicating that this habitat provides a stable niche for the microbial community (Jiang et al. 2021; Zhao et al. 2023a, 2023b). However, the fungal indicator groups were significantly enriched in NG (Figure 6D), with only the Rhodes indicator group being abundant in NG. It is generally accepted that, in a long‐term stable community (i.e., a community with minimal interference), a few highly competitive species tend to dominate the community (Doležal, Yakubov, and Hara 2013). Thus, the abundance of Rhododendron in NG further explains the decline in fungal diversity. Another contributing factor could be the difference in microbial functions between bacteria and fungi, particularly their ability to decompose organic and xenobiotic compounds. These findings indicate that bacteria and fungi play distinct roles in degraded grasslands.

### 4.3 Changes in Soil Microbial Community Function During Artificial Restoration of Degraded Grasslands in Black‐Soil Mountains

The results of FAPROTAX and FUNGuild suggest that similar to changes in community structure, bacteria and fungi form different functional microbial communities in various types of grasslands with significant differences. Different functional groups are often sorted into different environments that characterize the biochemical cycles in these regions (Crowther et al. 2019). Regarding the soil carbon cycle, AG increased soil bacterial chitinolysis and cellulolysis. This indicates an increase in the conversion of carbohydrates to alcohol or organic acids (Yu et al. 2021). In addition, AG significantly reduced the relative abundance of methylotrophy, indicating that soil bacteria rely more on carbon compounds, such as methanol or methane for energy. Regarding the soil nitrogen cycle, AG increased the relative abundance of aerobic chemoheterotrophy, ureolysis, chemoheterotrophy, and nitrate reduction. The results showed that the major metabolic groups in the soil microorganisms were influenced by AG. Compared with NG and BG, AG significantly changed the surface vegetation, leading to changes in root exudates that directly affected the microbial community and soil physical and chemical properties (Brackin et al. 2013). The major changes in the functional groups of the bacterial communities in AG were attributed to the combined effects of these differences.

Fungal functional groups have specific habitat adaptability; therefore, pathogenic and saprophytic fungi tend to thrive in nutrient‐rich environments (Nguyen et al. 2016). It was observed that ectomycorrhizal (EM) fungi exhibited a decreasing trend in AG, although the difference was not significant compared with NG and BG. In addition, the relative abundance of EM fungi, wood saprophytes, and plant pathogens decreased after the establishment of the artificial grassland. The reason for this result may be the interaction between the mycorrhizal fungi and free‐living microorganisms. These two groups have a complex interdependent relationship and both require nutrients for their growth and reproduction, leading to competition for resources (Orwin et al. 2011).

### 4.4 Carbon and Nitrogen Factors Affecting Soil Microbial Communities in the Process of Artificial Restoration of Degraded Grassland in Black‐Soil Mountains

Soil environmental factors are the primary drivers of the microbial community composition, diversity, and spatial distribution. However, various soil factors lead to significant differences in the composition, quantity, and diversity of soil microbial communities (Sun et al. 2022). A negative correlation was observed between bacterial diversity and total soil carbon (Figure 9A), which is consistent with the findings of Delgado‐Baquerizo et al. (2017). Soil TN plays a crucial role in affecting the microbial community, possibly because varying types of grassland plants result in different amounts, compositions, and decomposition rates of soil surface litter, thereby affecting the nutrient content of the soil surface (Chen et al. 2015). Soil TN is an important index of the nitrogen required for plant growth (Zhong et al. 2010), but it cannot directly meet the nitrogen demand of plants. Plants primarily uptake inorganic nitrogen, form root sheaths, and release root exudates, including amino acids, sugars, carboxylates, and secondary metabolites. These components can provide rich nitrogen sources for soil microorganisms, thereby enhancing the activity and diversity of microorganisms and changing their species composition (Zhang et al. 2013). The levels of soil ${\mathrm{NH}}_{4}^{+}-N$, ${\mathrm{NO}}_{3}^{-}-N$, BG, and MBC were significantly associated with changes in the α‐diversity of bacteria, whereas the contents of TC, TN, LAP, and MBN were significantly related to changes in the α‐diversity of fungi (Figure 9B). It can be seen that the changes in soil physical and chemical properties caused by the artificial planting of degraded grassland in black‐soil mountains are the main driving factors for the changes in the α‐diversity index and composition of soil microorganism communities. One possible reason for this is that after 5 years of vegetation restoration, the α‐ and β‐diversities of the soil bacterial and fungal communities were significantly different. This indicates that, under the influence of different dominant species, the response of microbial community diversity changes differently, and different species may affect the formation of distinct microbial communities. This effect is evident in the changes in the species composition and relative abundance of microorganisms. This finding is consistent with recent findings on soil microbial diversity in relation to plants. Microbial diversity is primarily regulated by aboveground or dominant species (Rinella, Espeland, and Moffatt 2016). After planting AG, soil resources and plant communities changed, leading to an increasing similarity between soil bacteria and fungi groups and healthy grasslands. In addition, the functional groups of bacteria and fungi in the AG were more similar to those in healthy grasslands, supporting the view that soil microbial function can indicate a recovery trajectory (Hart et al. 2020). The correlation between microbial community structure and soil properties further validates that the microbial community structure can be used to evaluate the soil environment (Banning et al. 2011). According to the results of the RDA (Figure 10), it was inferred that most carbon and nitrogen factors were related to the abundance of soil bacterial and fungal communities and functional groups. This is consistent with the results of Jiang et al. (2021), who concluded that microbial communities are significantly correlated with soil nutrients. In addition, soil ${\mathrm{NH}}_{4}^{+}-N$, ${\mathrm{NO}}_{3}^{-}-N$, TC, TN, BG, LAP, MBC, and MBN were driving factors affecting bacterial and fungal communities. Among these, TN, TC, and LAP were the most significant, whereas BG was the main factor affecting the fungal functional groups. Different grassland types may have varying ground coverage, leading to changes in litter accumulation, root exudates, and soil nutrients, resulting in variations in microbial species and quantities, which subsequently affect soil enzyme activities (Pommier et al. 2018). This finding is consistent with the results of Guo et al. (2019), suggesting that soil carbon and nitrogen, as the energy suppliers of soil microorganisms, are important factors influencing changes in soil enzyme activity. Higher soil carbon and nitrogen content lead to more active soil microorganisms, indicating that the change in the microbial community depends on the supply of soil nutrients (carbon and nitrogen). This finding is consistent with the conclusions of McGee et al. (2019) that different forms of inorganic nitrogen may significantly affect soil microbial biomass and community composition. The artificial restoration process leads to changes in the vegetation community, subsequently affecting the plant litter quality and related matrix components. Notably, changes in the soil characteristics caused by black‐soil mountain grassland affected the soil microbial community and its functional structure, although these changes were not uniform.

## 5 Conclusions

Artificial planting significantly increased the soil total carbon, total nitrogen, ammonium nitrogen, microbial biomass carbon, microbial biomass nitrogen, and leucine aminopeptidase content. It also significantly affected the diversity and composition of soil bacteria and fungi in the process of artificial restoration of degraded grassland in black‐soil beach, but had little effect on fungal diversity. This indicates that the response of the soil bacterial community to artificial planting was stronger than that of the fungal community in this study. Ammonium nitrogen, nitrate‐nitrogen, β‐1,4‐glucosidase, and microbial biomass carbon were positively correlated with bacterial α‐diversity. Total carbon, total nitrogen, leucine aminopeptidase, and microbial biomass nitrogen were positively correlated with fungal α‐diversity. Artificial planting has a significant impact on the function of the soil bacterial community, especially the soil bacterial community related to soil carbon and nitrogen cycles. Soil total carbon, total nitrogen, leucine aminopeptidase, and ammonium nitrogen were the key carbon and nitrogen factors affecting microbial community structure. Ammonium nitrogen and β‐1,4‐glucosidase were the main carbon and nitrogen factors, respectively, that affected functional changes in fungi. In addition, specific bacteria (Acidosporium and Mycobacterium verrucosum) and fungi (Ascomycetes and Basidiomycetes) can be used as biomarkers for artificial restoration processes. In summary, these results strengthen our understanding of the relationship between microbial communities, microbial functions, and soil characteristics in the process of artificial restoration of degraded black‐soil mountain grasslands. They provide a basis for further understanding of microorganisms in grassland vegetation restoration on the Qinghai–Tibet Plateau. Artificial planting has a positive effect on restoring degraded mountain grasslands. However, after artificial planting, attention should be paid to its management and protection. A reasonable grassland utilization system should be formulated to ensure sustainable grassland utilization.

## Author Contributions

**Lele Xie:** conceptualization (equal), data curation (equal), investigation (equal), software (equal), writing – original draft (equal). **Yushou Ma:** data curation (equal), funding acquisition (equal), project administration (equal), resources (equal), writing – review and editing (equal). **Yanlong Wang:** investigation (equal), resources (equal), writing – review and editing (equal). **Yuan Ma:** data curation (equal), investigation (equal), writing – review and editing (equal). **Xiaoli Wang:** methodology (equal), supervision (equal), writing – review and editing (equal).

## Conflicts of Interest

The authors declare no conflicts of interest.

## Data Availability

Hereby affirm that primary data including total data will be deposited in the Dryad Repository when the paper is accepted. Dryad: https://doi.org/10.5061/dryad.kkwh70sc7. The data that support the findings of this study are available from the corresponding author upon reasonable request. The data will be stored in https://datadryad.org/stash/share/1hZR1_biGB‐afckDcIS1WFbsbYwZw3d3‐RPlXWZgTTs.
